# 

**Published:** 1889-09-21

**Authors:** 


SATURDAY, SEPTEMBER 21st, 1889.

**The Public Servants of the Future** - - - - - 383

**Words of Consolation**—The Tenderness of Christ - - 384

**The Doctor's Pulpit.**—Quiet Habits: Long Life- - - 385

**Editor's Letter-Box.**—Overcrowded Cemeteries—Hotels and their Critics - - - - - - - - - - - 386

**"Left-Leggedness."** By DR. W. K. SIBLEY - - - - 387

**Children's Country Holidays Fund** - - - - - 387

**The Boarding Out of Pauper Children.** II.—Miss Mason's Report - - - - - - - - - - - 388

**"Education by Picnic"** - - - - - - - - - - 389

**Notes and News** - - - - - - - - - - 390

**Everybody's Page** - - - - - - - - - - 392

**Annotations.**—Asylums or Hospitals?—Dogs and Muzzles —Atraperna's Neighbour - - - - - - - - - 393

**Her Heart's Mistake.** By E. D. CUMMING. Chaps. xxiii. and xxiv. - - - - - - - - - - - - - 394

**Mandragora** - - - - - - - - - - - 397

**Amusements—Scraps and Gleanings** - - - - - 398

**EXTRA SUPPLEMENT—THE NURSING MIRROR.**

**En Passant.**—Our Practical Queen—Evelina Hospital—Nurse or Doctor—Midwifery in America—Stratford-on-Avon—Overcrowding—Heroism - - - - - - - - xcv
**Physiology for Nurses.** No. 10.—The Mechanism of Movement - - - - - - - - - - - - -xcvi
**More Voyages in Vacation.**—I. - - - - - - -xcvi
**Everybody's Opinion.**—The Pension Fund—Outdoor Uniform—Examination Questions—Asylum Attendants - xcvii
**Appointments** - - - - - - - - - - - xcviii
**Vacancies—Notes and Queries** - - - - - xcviii

